# Pyorrhœa Alveolaris Infection

**Published:** 1886-01

**Authors:** A. W. Harlan


### Pyorrhœa Alveolaris Infections.—Alveolitis, Phagdenic Pericementitis.

### SYMPTOMS.

Incipient Pyorrhœa may be observed at any age after the attainment of the teeth. The teeth most frequently affected are the upper incisors, bicuspids, first and second molars, lower molars, second bicuspids and incisors, blunting of the septum of gum between the teeth, detachment of the pridental membrane at the gingival margin, partially or completely encircling the tooth—formation of a pocket or pockets, of varying depths, the gum presenting a slightly reddened, sometimes thickened appearance, deep red or purplish outlining the pocket, wasting of the alveoli on the labial and lingual surfaces of the teeth, deposits of serumal calculi on the surfaces of the roots beneath the detached gum, pus exuding on pressure, teeth become loose and drop out, or become irregular. Salivary calculus may be associated with Pyorrhœa, but is easily distinguished from it by the gums bleeding freely, when it is removed and the quick subsidence of the inflammation, and the rapid re-attachment of the gums to the teeth.

### ETIOLOGY AND PATHOLOGY.

Any injury of the dental ligament, as the extraction of an adjacent tooth, rapid wedging, forcing rubber bands, cloth or ligatures around the teeth, injudicious use of tooth picks, the use of clamps over the teeth to retain the rubber dam, salivary deposits, (rarely) lack of cleanliness, use of brushes too stiff and not properly constructed, hereditary tendencies, or any inflammation of the gums sufficient to detach the peridental membrane, furnishing a spot for a *nidus*, around which may be deposited serumal or sanguinary calculus. Such careful observers as Witzel, Arkovy, Islai, Black, and others, state that Pyorrhœa is a specific infectious disease, proliferated by a micrococcus not yet classified, which may always be found in the pus exuding from the pockets. It is believed to stand in a causul relation in its inception.

Pyorrhœa may and does exist independently of calcic inflammation. Numerous cases have been observed where salivary deposits coated the teeth of one portion of the mouth and other teeth in the same mouth were affected by Pyorrhæa.

### TREATMENT.

Until recently, dental surgeons have almost despaired of a successful treatment. At present it is claimed that many teeth formerly lost, may be saved. All deposits are removed by the use of thin flat scalers without mutilating the gums; this is accomplished by a pushing motion, the edges of the alveoli are excised or scraped, and the pockets syringed one at a time with per oxide of hydrogen to cleanse them of debris and destroy bacteria. After which solutions in water of iodide of zinc, grs., vi., xii., xxiv., or saturated aqueous solution of tartrate or carbolic acid crystals 2 parts oil wintergreen 3 parts, oil cinnamon 1 part, are to be injected into the pockets twice per week, to stimulate the formation and growth of new tissue around the root of the tooth. Persistency in this treatment, it is confidently asserted, will lead to a cure. In case the teeth are very loose or out of line, they should be wired together with fine binding wire pending treatment.

---

### DISCUSSION.

Dr. Harlan. I know of no agent that will so thoroughly clean out the pockets as Per Oxide of Hydrogen; it does this by effervesence.

Dr. Watt. Is it not the nascent Oxygen that is liberated, which does the work?

Dr. Harlan. Yes sir. There are two classes of organisms, one needs Oxygen to support its life, the other is destroyed by it; the organisms of Pyorrhœa Alveolaris belong to the latter, and that is the reason Per Oxide of Hydrogen is the best remedy to use in the cleansing of the pockets, the germs being distroyed at the same time.

Dr. Rehwinkel. There is nothing new in the way of additional phases of the disease; observations have not led me to deviate from the views I formed ten or twelve years ago, and I now see no reason to change them. At that time I met with much antagonism, and found but few who would agree with me; my friend, Dr. Rawls, is one of the few.

At that time there was no distinct name for the disease, pupils of Dr. Riggs appropriated it all, and called it "Riggs' Disease." Some time ago, when I was to read a paper on the subject before the American Dental Association, I wrote to Dr. Riggs, asking him if he considered the disease constitutional or merely local; I received no reply. I was at a loss to determine his position, but was led to believe that Dr. Riggs claimed the disease was entirely local; he holds that view to-day.

Myself and Dr. Chase, of St. Louis, were the first to suggest that the deposits of Pyorrhœa Alveolaris were not those of the saliva, and, furthermore, were not local in their nature. Dr. Ingersoll calls it Sanguinary Calculus, while Dr. Black terms it Serumal.

During the first agitation of the subject, it was difficult to determine and distinguish what was meant and to differentiate a true case of Pyorrhœa Alveolaris, distinct from an inflammation of the gum, incident upon accumulations of salivary calculus. The definitions of European writers (more especially the Germans) were far more exact in their nomenclature; Magitot may have used the term Pyorrhœa Alveolaris at that time. Since then, the best place to get a true idea is in the North of Ireland and Scotland, as there, the teeth are good, sound, free from decay, and consequently exhibit the best specimens of loss of the teeth from this disease.

I cannot get rid of the idea of a co-incident or co-equal loss of the teeth from this cause, with loss of the hair; the idea is almost a conviction.

Great and wonderful progress has been made in this direction during the last ten years, and pre-eminent amongst others, stand Drs. Rawls and Harlan. Dr. Harlan has given us the best course of treatment, while Dr. Rawls has brought out many points concerning the cause, especially the effects of salt and mercury in producing predisposing causes together with heredity.

We do not treat as heroically now as we did five years ago, milder measures are employed, and it is found that treatment once or twice a week is just as effective as daily treatment, the latter being productive of no especial good. Dr. Riggs has always regarded Pyorrhœa Alveolaris as a local affection merely, and was unfortunate enough to remark at the Inter-National Medical Congress at its meeting in London, that he could produce the disease at will every time by merely placing a rubber band around the neck of the tooth and he would have the disease manifest itself in three or four days.

Dr. Watt. I wish to emphasize the avoidance of over treatment. I think I was the first to advocate pressure upon the gums with the fingers; the pressure acts as a stimulant, the object being to press the stagnant blood out of the engorged capillaries, new blood then passes into them and tones them. Continued pressure several times a day, will act as a tonic upon the vessels so that they become able to empty themselves, the circulation being restored. Dr. Harlan's paper is worth any seventeen papers, except Dr. Rawls', that I have ever heard. Nothing is so beautiful as a clear-cut thought, it is more beautiful than a humming bird. I have frequently been called "Old Nascent" because I insist that elements act better in their nascent than in their passive state. Bright iron may be left in oxygen for an indefinite length of time without rusting, but, pass the electric spark through the gas, ozone will be formed with the result that the iron will be eaten up very quickly. Dr. Harlan's theory as to Peroxide of Hydrogen ($H_2$ $O_2$) is correct. The remedy is just the one you need as the compound cannot retain the extra quantity of oxygen when it comes in contact with the least bit of organised matter, as the oxygen is immediately given up in its nascent condition, and the micro-organisms are burnt up alive. This oxygen is not the oxygen we require for breathing, it would destroy us. Even in a minute proportion in the atmosphere, due to prolonged, high electrical tension, it produces a change in the atmospheric oxygen and causes irritation and bronchial troubles. I do not think that mercurial treatment is a leading cause of disease. I am inclined to think that Dr. Riggs was a little self-important in his treatment of the disease. "Incrustations of Salivary Calculus," Dr. Morgan said at the meeting of the American Dental Association, at Minneapolis, "is an evidênce of too much lime in the system." In the healthiest people I could find, I have always found the two forms of lime in their saliva, and have concluded they were normal constituents. They are held in solution in the saliva by reason of free carbonic acid, and when that is driven off, the lime is precipitated. If there is too little or too much lime in the saliva, or if there is any, as soon as you take away the carbonic acid, the lime will be precipitated. If there is ammoniacal degeneration and formation of carbonate of ammonia, the lime will be precipitated. Serumal deposits, so far as I am able to determine, contain both the carbonate and phosphate of lime, though no organic matter.

Dr. H. A. Smith. If micro-organisms are present and produce the disease, then a germicide is indicated, if the germ is pathogenic; if it is a septic organism, then some other form of anticeptic is required. It is nonsense not to accept the theory that a micro-organism is a cause, merely because you may not have seen it. Pasteur and others are authority that there are such organisms; if this is so, the disease *can* be infectious and inoculation take place from one pocket to another. If the organism is of septic origin, per oxide of hydrogen will be of no use; if merely septic, cleanliness is all that is indicated, as the trouble is, in many mouths there is a great deal of filth and germs of all kinds.

I do not regard Pyorrhœa Alveolaris as a systemic or hereditary disease, I admit that you have a hereditary impression, but I do not know *why*. There is a diathesis, an idiosyncracy, a tendency that makes one take a disease and another one not take it. I do not know *why* I look like my father, any more than Dr. Rawls does.

Dr. Rawls. The gentlemen who have just spoken have made me say that mercury and salt are *causes* of the disease. I did *not*. They merely produce conditions that will render them susceptible to the acquirement or contraction of the disease. I

hold that the color of the eye, the hair, and the form of the cells in the brain are produced by an impression made upon them by the food taken into the system, climatic influences and other circumstances and surroundings included in what we term environment. These impressions stamp themselves upon the various tissues and the cells composing them, and become an integral part of the individual. It is this that makes me look like my father and think as my father thought.

Dr. Watt. I see no use for loud and excited talk; take things calmly.

Dr. J. Taft. Dr. Rawls is not mad, I have heard him talk that way before.

Drs. Watt and Rawls both mentioned the advantage of manipulating the gums by pressure upon them, with the fingers for the purpose of inducing a healthy circulation in the capillaries. That is correct, but it can be carried further; when the gums and teeth are sore, there is an indisposition to use the teeth, but it must be done to press out the blood and so increase the activity of the circulation, and prevent an accumulation of effete matter in the blood. In addition to all the methods of treatment mentioned, and which are good, thorough exercise of the teeth by mastication, must be carried out to induce a rapid and frequent interchange of blood in the vessels. This is evidenced by the good condition of the teeth upon one side of the mouth that has been thoroughly used, while those of the other side not in use exhibit all the symptoms and characteristics of disease. Pyorrhœa Alveolaris is not manifested first in the gum, but in the periosteum, then extending to the tissues of the gums.

Dr. Harlan. I have been somewhat pained and amused at times by practicing dentists who pointed to simple aggregations of tartar as Pyorrhœa Alveolaris, and I have been called in consultation to see cases in which salivary calculus on the lower incisors was pointed out as the true cause of Pyorrhœa, and where there had been prolonged medication without a cure, because the deposits had not been removed.

Ordinary dental tartar is *not* a cause of Pyorrhœa Alveolaris; that you may have an inflammation of the gums about it there is no doubt, but below the tartar, the periosteum is firmly adherent and no one ought to fail to discern it. These cases that will get well without treatment are the ones that are paraded in the prints as Riggs' Disease, cured on one treatment.

Drs. Watt and Rehwinkel. It is Riggs' Disease.

Dr. Harlan. It is not contended by any one that serumal deposits are a *cause*; the deposits of this kind are a result of the disease. I am a believer in the germ theory of the disease, and I state it frankly, but you must have an injury to the parts first. I do not believe in heredity. I have seem many adults with the disease and their children do not have it, though they also were adults. I have numerous specimens that were so loose as to be pulled out with the fingers where the little islands of deposits were not at the gingival margin, but down between the apex and the gum line, sometimes upon the apex of the root. A great deal is said about the thorough removal of these deposits, but it is not always done on account of the pain, great difficulty of access and lack of proper instruments. In all these cases the periosteum is *always* detached. In removing the deposits, instead of making a vertical incision or slit of the gum, the incision is made below the gingival margin and parallel with it; this, when healed, does not leave a puckering or depression of the gum. It is extremely important after loosening the deposits, to get them out, as they might just as well be left on the root as loose and wedged in, the result being a supperative inflammation.

It is a mistake to try to clean too many teeth at one sitting; it is better to be sure that you have thoroughly cleansed one tooth at a time and get the deposit out of the pocket. If anticeptics and germicides are not used at least once in four days, the spores will be found to fill the pockets.

Dr. Smith. Will you please state whether the spories are septic or not?

Dr. Harlan. From many conversations with Dr. Black and others, I have come to the conclusion that they are pathogenic, so that the disease may be produced in another pocket by inoculation. The spore must have a certain condition of the parts to manifest itself; one observer has stated that he has produced the disease by inoculation in the mouth of a dog. The spore must have a place to grow in. If we cannot answer all questions that may arise, it is no disgrace to us, as our observations are but in their infancy.

Dr. Smith. Is the disease like a catarrh?

Dr. Harlin. We have buccal and nasal catarrh, in the latter there is a calcareous deposit in the plate, but it is not of the consistence of those on the roots. In nasal catarrh there is more of a putrefactive process, the result of which frequently excoriates the openings of the nostrils, makes the parts sore, etc., while in Pyorrhœa Alveolaris, we cannot see these conditions. I will not go so far as to state that I can produce new alveolar process and tissues, though in many cases, loose teeth become tightened. These teeth, pending treatment must be held firm and steady, with suitable ligatures. It is not necessary to continue treatment after pus has ceased to flow.

Dr. Smith. The question of Dr. Rawls is very interesting; if the disease is induced by septic germs, why can it not be produced in another mouth where there is a break, but the disease is not acquired? The conditions are not the same, but I contend that it is contagious and can be transmitted.

Dr. Rawls. It is the condition the body is in that renders it susceptible to the acquirement of the disease. The spores and micro-cocci *follow*.

Dr. Smith. Take the case of erysipelas in a hospital, it is specific; in one ward many patients will have it and under like conditions in another ward none will get it.

Dr. Rawls. Puerperal fever can be transmitted by a specific germ, and was carried across the yards in King's College Hospital, London, in the clothes of attendants and others.

The condition or susceptibility must exist before Pyorrhœa Alveolaris may manifest itself. There must be a bruise or injury to the gum, from tartar, toothpick, or what-not, *then* comes on the inflammatory action, then the loosening of the gum *below* the gingival margin, even while the latter remains firmly attached; the process of wasting is started, the parts succumb and melt away.

When a tissue is deprived of its nutrition, all dead parts must be cut away, down to a point where you will get the nutrient currents of supply, not of blood alone, but the anastomosis of capillaries where you get nutrition by endosmotic action, *then* the gum will drop down to the bone. It is absurd to apply iodine to induce the gum to *crawl* up upon the tooth.

*No* course of treatment will produce a cure without a re-establishment of the nutrient currents.

Dr. C. R. Butler. I am very much pleased with Dr. Harlan's method of incision instead of making a slit. If the teeth are made comfortable for a few years, we have done the patient much good. The less of the gum tissue destroyed, the better.

Dr. Rehwinkel. Is this disease produced by a specific germ or not?

Ten years ago it was thought serumal deposits were a consesequence, and now it is said that micro-organisms are a sequence.

Heredity has been made light of, but, if all departments of science were as well established, we would be much the wiser. I don't doubt that micro-organisms are found in the pockets, but they are a mere concomitant, and it is just upon this point that will hinge the thoughts for the next five years."

Adjourned.

Mr. Beer then read a paper on Dental Literature.

SECOND DAY—EVENING SESSION.

The meeting was called to order by President James, at 7:45, the minutes of previous session were read and approved. A motion was carried to make the final adjournment of the Society at 11 o'clock Friday morning.

A motion to go into the election of officers prevailed.

Dr. Betty proposed for honorary membership, Drs. A. O. Rawls, of Lexington, Ky.; A. W. Harlan, of Chicago, Ills.; Wm. Van Antwerp, of Mt. Sterling, Ky., and W. W. Justus, of Winchester, Ky.

Dr. Keely proposed Drs. A. A. Blount and N. W. Williams, both of Geneva, Switzerland. Dr. Smith proposed Dr. W. D. Miller, of Berlin, Prussia. The rules were suspended and the gentlemen elected by the Secretary's ballot.

The election of officers for the ensuing year, is as follows:
President, F. H. Rehwinkel, Chillicothe.
First Vice-President, H. H. Harrison, Cadiz.
Second Vice-President, N. S. Hoff, Cincinnati.
Recording Secretary, J. R. Callahan, Hillsboro.
Treasurer, G. W. Keely, Oxford.

BOARD OF DIRECTORS.

*For three Years.*

H. A. Smith, Cincinnati.
J. Taft, Cincinnati.
C. H. Harroun, Toledo.
A. F. Emminger, Columbus.

*For two Years.*

D. R. Jennings, Cleveland.
C. R. Butler, Cleveland.
F. A. Hunter, Cincinnati.
W. R Lilly, Circleville.

*For one Year.*

E. G. Betty, Cincinnati.
F. C. Runyan, Springfield.
M. H. Fletcher, Cincinnati.
L. B. Welch, Wilmington.

The balloting for members of the Dental Examining Board, resulted in the choice of Drs. C. R. Butler and E. G. Betty.

The bill of Secretary Callahan, $32.45 for stationery, books, etc., was ordered paid. The Secretary and Treasurer were each paid $25.00. The Janitor, $5.00.

On resolution of Dr. Rehwinkel, a message of sympathy was sent to Dr. I. Williams, he being very ill and unable to attend.

A standing vote of thanks was tendered the Mayor and City Council of Chillicothe for their courtesy in giving the use of the Council Chamber to the Society.

On motion of Dr. Keely, the papers and other documents for publication were turned over to the *Ohio State Journal* and the Register. Adjourned.

THIRD DAY—LAST SESSION.

The meeting was called to order by President James promptly at 9 o'clock, A. M., the minutes of the previous day's sessions were read and approved.

Vice-President Harrison now took the Chair.

On motion of Dr. James, the Secretary was instructed to print 500 copies of the Constitution and By-Laws.

Dr. Betty then read a paper, for which see page 14.

On motion, the Chair appointed Drs. E. G. Betty, N. S. Hoff and C. R. Butler a Committee on Statistics, to which Committee the matter was referred with power to act, and report at next annual meeting.

Dr. J. Taft then read a paper on Association Work ; see page 5.

Dr. James read the paper of Dr. E. Parsons, of Savannah, Ga. ; see page 19.

Dr. Watt then read an editorial prepared for the November number of his journal.

Dr. C. R. Butler then read his paper on—

